# Rapid Quality Improvement Project (RQIP): Analysis of All Referrals to the Newcastle Psychiatric Liaison Team (PLT) From the Emergency Department (ED) During April 2023

**DOI:** 10.1192/bjo.2024.425

**Published:** 2024-08-01

**Authors:** Eleanor Romaine, Harriet Flude, Katie Taylor, Sarah Brown, Isabella Donnelly

**Affiliations:** Cumbria, Northumberland, Tyne and Wear NHS Foundation Trust, Newcastle upon Tyne, United Kingdom

## Abstract

**Aims:**

In light of increasing referral rates, the RQIP aimed to review all referrals made to PLT by the ED during April 2023. The purpose was to identify ways to improve working practices to benefit patients, the ED team and the PLT.

**Methods:**

All ED referrals in April 2023 were identified and the following was gathered from each record.

*Patient information:*

1. Record number, sex, age

*Circumstances of attendance:*

1. Date/time
2. Who directed patient to ED and arrival method
3. Attendance reason
4. Presence/absence of physical health condition requiring ED
5. Intoxication on arrival and if assessment required after sobriety
6. Outcome

*Patient involvement with other services:*

1. Number of previous PLT referrals in past 90 days
2. Currently under care of another team, if yes, were they contacted before ED attendance
3. Contact with crisis team in previous 72hours

**Results:**

During April 2023 there were 356 referrals from ED to PLT. 284 represented single attendances and 72 represented repeat attendances by 44 patients. 34% (n = 123) self-presented to ED. Emergency services directed 21% (n = 75) to ED. 71% (n = 253) had physical health reasons to attend whilst the rest presented with mental health crisis alone (n = 103). 25% of patients attending ED and referred to PLT were intoxicated and a third of these did not require assessment following sobriety. 41% (n = 145) patients were open to another mental health team within the trust who could potentially have provided crisis input. Of all referrals 27% (n = 97) were signposted to other services, 26% (n = 93) left before they were seen. PLT referred 11% (n = 40) to crisis teams and 3% (n = 11) to Mental Health Act assessment.

**Conclusion:**

Findings indicate that a large proportion of patients attending ED could have had their mental health needs met elsewhere in the absence of a medical reason for attending, thus potentially avoiding long waits in ED. Patients that are referred but leave before assessment, those without acute medical need to be in ED, those that do not require assessment after sobriety or those open to other planned care mental health teams may have their needs best met outside of the acute ED environment. It is hoped that community transformation work will enable community services to become more responsive to such needs.

The team propose working collaboratively with the acute trust and trialling embedding a PLT clinician in the ED triage process in order to redirect patients to the most appropriate care in a timely way.

